# 2-{(*E*)-[(2*Z*)-2-(1,2-Di­hydro­phthalazin-1-yl­idene)hydrazinyl­idene]meth­yl}phenol

**DOI:** 10.1107/S1600536813024203

**Published:** 2013-09-07

**Authors:** M. K. Prasanna, M. Sithambaresan, K. Pradeepkumar, M. R. Prathapachandra Kurup

**Affiliations:** a Department of Chemistry and Research Centre, PRNSS College, Mattanur 670 702, Kannur, Kerala, India; b Department of Chemistry, Faculty of Science, Eastern University, Sri Lanka, Chenkalady, Sri Lanka; c Department of Applied Chemistry, Cochin University of Science and Technology, Kochi 682 022, India

## Abstract

The title compound, C_15_H_12_N_4_O, adopts an *E* conformation with respect to the azomethine bond and crystallizes in its hydrazinyl­idene tautomeric form. The dihedral angle between the ring systems is 15.98 (7)°. The phenol O—H group forms an intra­molecular O—H⋯N hydrogen bond. In the crystal, pairs of N—H⋯N and C—H⋯O hydrogen bonds link neighbouring mol­ecules into centrosymmetric dimers. These dimers are inter­connected by means of three types of π–π stacking inter­actions. One, with a centroid–centroid distance of 3.577 (1) Å [inter­planar separation = 3.4673 (6) Å], connects adjacent mol­ecules into centrosymmetric dimers. The other two inter­actions, on the outward facing sides of the dimers, are between phenol rings of neighboring mol­ecules [centroid–centroid separation = 3.7907 (13) Å and inter­planar separation = 3.5071 (8) Å], and between phthalazin units [centroid–centroid separation = 3.6001 (12) Å and inter­planar separation = 3.4891 (7) Å]. In combination, the π–π inter­actions lead to the formation of infinite layers with mol­ecules stacked along [0-11]. These layers are, in turn, connected with neighbouring layers through the N—H⋯N and C—H⋯O hydrogen bonds, yielding a three-dimensional supra­molecular architecture.

## Related literature

For biological properties of phthalazine and its derivatives, see: Awadallah *et al.* (2012 ▶); Minami *et al.* (1985 ▶); Zhang *et al.* (2010 ▶); Bian *et al.* (2013 ▶). For applications of 1-phthalazinyl hydrazones in optoelectronics, see: Caruso *et al.* (2005 ▶). For the synthesis of related compounds, see: El-Sherif *et al.* (2012 ▶). For related structures and background references, see: Shafiq *et al.* (2013 ▶).
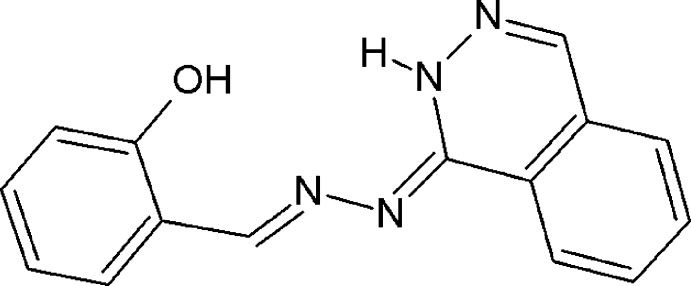


## Experimental

### 

#### Crystal data

C_15_H_12_N_4_O
*M* *_r_* = 264.29
Triclinic, 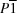

*a* = 6.8028 (12) Å
*b* = 8.4263 (13) Å
*c* = 11.868 (2) Å
α = 89.774 (9)°
β = 83.113 (9)°
γ = 70.356 (8)°
*V* = 635.62 (19) Å^3^
*Z* = 2
Mo *K*α radiation
μ = 0.09 mm^−1^
*T* = 296 K
0.25 × 0.20 × 0.20 mm

#### Data collection

Bruker Kappa APEXII CCD diffractometer
Absorption correction: multi-scan (*SADABS*; Bruker, 2004 ▶) *T*_min_ = 0.978, *T*_max_ = 0.982
3781 measured reflections
2204 independent reflections
1623 reflections with *I* > 2σ(*I*)
*R*_int_ = 0.020

#### Refinement

*R*[*F* ^2^ > 2σ(*F* ^2^)] = 0.041
*wR*(*F* ^2^) = 0.117
*S* = 1.03
2204 reflections
175 parameters
2 restraints
H atoms treated by a mixture of independent and constrained refinement
Δρ_max_ = 0.16 e Å^−3^
Δρ_min_ = −0.17 e Å^−3^

### 

Data collection: *APEX2* (Bruker, 2004 ▶); cell refinement: *APEX2* and *SAINT* (Bruker, 2004 ▶); data reduction: *SAINT* and *XPREP* (Bruker, 2004 ▶); program(s) used to solve structure: *SHELXS97* (Sheldrick, 2008 ▶); program(s) used to refine structure: *SHELXL97* (Sheldrick, 2008 ▶); molecular graphics: *ORTEP-3 for Windows* (Farrugia, 2012 ▶) and *DIAMOND* (Brandenburg, 2010 ▶); software used to prepare material for publication: *SHELXL97* and *publCIF* (Westrip, 2010 ▶).

## Supplementary Material

Crystal structure: contains datablock(s) I, global. DOI: 10.1107/S1600536813024203/zl2563sup1.cif

Structure factors: contains datablock(s) I. DOI: 10.1107/S1600536813024203/zl2563Isup2.hkl

Supplementary material file. DOI: 10.1107/S1600536813024203/zl2563Isup3.cml

Additional supplementary materials:  crystallographic information; 3D view; checkCIF report

## Figures and Tables

**Table 1:** Hydrogen-bond geometry (Å, °)

*D*—H⋯*A*	*D*—H	H⋯*A*	*D*⋯*A*	*D*—H⋯*A*
N3—H3′⋯N4^i^	0.89 (1)	2.31 (1)	3.0181 (14)	137 (2)
O1—H1*A*⋯N1	0.85	1.89	2.6362 (15)	147
C15—H15⋯O1^i^	0.93	2.59	3.224 (3)	125

Symmetry code: (i) 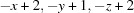.

**Fig. 1.**
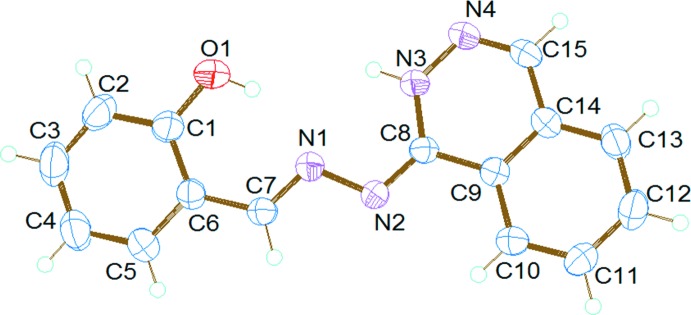
ORTEP view of the compound, drawn with 50% probability displacement ellipsoids for the non-H atoms (the minor moiety H atom was omitted for clarity).

**Fig. 2.**
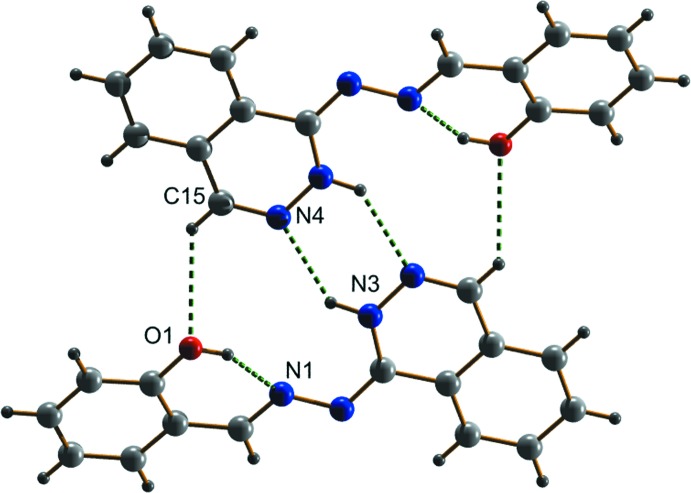
Graphical representation showing the centrosymmetric dimers by means of hydrogen bonding in the crystal structure of C15H12N4O. The minor disordered H atoms were omitted for clarity.

**Fig. 3.**
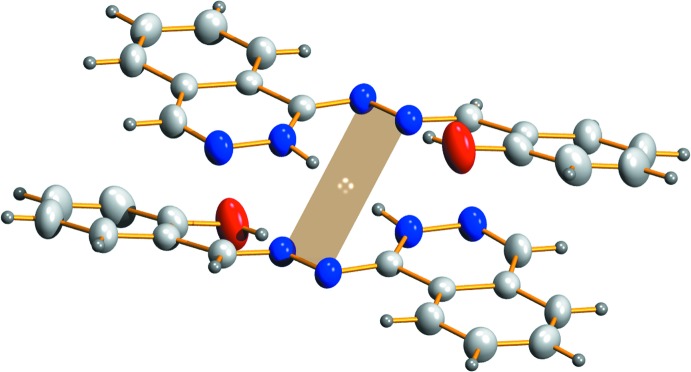
Graphical representation showing π–π interactions between whole molecules into centrosymmetric dimers in the crystal structure of the title compound.

**Fig. 4.**
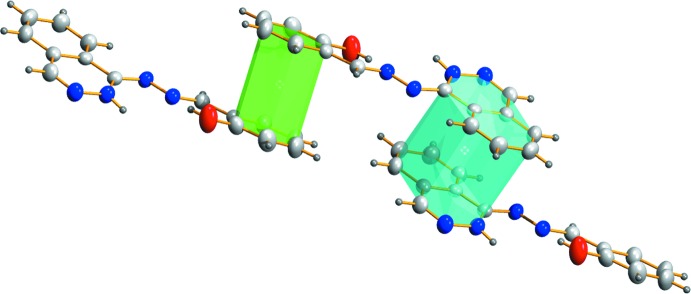
Graphical representation showing π–π interactions between phenol and pthalazin rings in the crystal structure of the title compound.

**Fig. 5.**
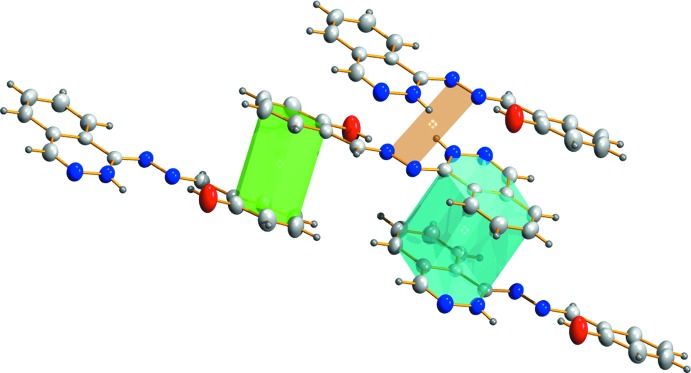
Graphical representation showing π–π interactions that lead to formation of infinite layers in the crystal structure of the title compound.

**Fig. 6.**
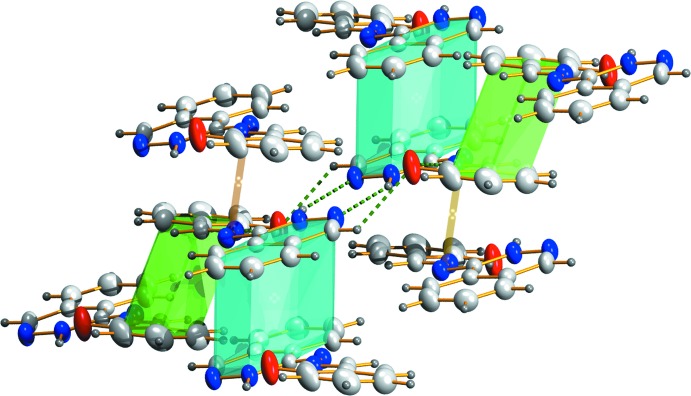
Graphical representation showing neighboring layers formed by π–π interactions and connected through intermolecular N–H···N and C–H···O H-bonds

**Fig. 7.**
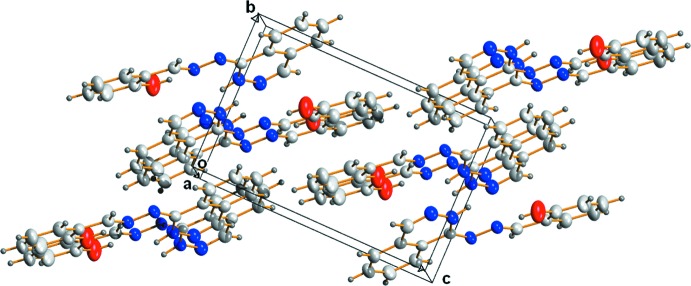
Packing diagram showing the molecular assembly of the title compound along the a axis.

## supplementary crystallographic information

### 1. Comment

Hydralazine, or 1-hydrazinylphthalazine, is a direct-acting smooth muscle relaxant used to treat hypertension by acting as a vasodilator, primarily in arteries and arterioles. Upon condensing with carbonyl compounds hydralazine will form hydrazones, namely 1-phthalazinyl hydrazones, which find use as vasodilating antihypertensive drugs and also application in optoelectronics (Caruso *et al.*, 2005).

The title compound is one such 1-phthalazinyl hydrazone. It crystallizes in the triclinic, *P*1, space group. The molecule exists in its *E* configuration with respect to the C7=N1 bond which is confirmed by the torsion angle of 177.11 (12)° of the C6—C7—N1—N2 moiety (Fig. 1). The torsion angle of -5.33 (17)° of the N1—N2—C8—N3 moiety shows that the N1 and N3 atoms are *cis* to each other. The C7=N1 [1.2859 (16) Å] and C8=N2 [1.3010 (16) Å] bond distances are very close to the formal C=N bond length of reported similar compounds [C=N; 1.282 (4) and 1.288 (3) Å, respectively] (e.g., Shafiq *et al.*, 2013), confirming the azomethine bond formation and the presence of a hydrazinylidene. The phenol, azomethine and phthalazin moieties are nearly planar (rms deviations 0.0041, 0.0000 and 0.0328 Å respectively) and coplanar to each other, with the two moieties at the ends of the molecule slightly twisted away from the central moiety in opposite directions by torsion angles of 7.67 (10) and 8.68 (11)° for the phenol and phthalazin moieties with the central azomethine moiety, respectively. The dihedral angle between phenol and phthalazin moieties is 15.98 (7)°.

The phenolic O–H group forms an intramolecular O–H···N hydrogen bond with a D···A distance of 2.6362 (15) Å, and two intermolecular N–H···N and C–H···O hydrogen bonding interactions are found between the neighbouring molecules with D···A distances of 3.017 (2) and 3.224 (3) Å. These intermolecular hydrogen bonds operate together to form centrosymmetric dimers in the crystal lattice. These dimers are interconnected by means of three types of π–π stacking interactions. One of them connects whole molecules into centrosymmetric dimers with a centroid to centroid distance of 3.577 (1) Å (interplanar separation: 3.4673 (6) Å) (Fig. 3). The other two, on the outward facing sides of the π-stacked dimers, are between phenol rings of neighboring molecules (centroid-centroid 3.7907 (13), interplanar separation: 3.5071 (8) Å), and between phthalazin moieties (centroid-centroid 3.6001 (12), interplanar separation: 3.4891 (7) Å) (Fig. 4). The π–π interactions lead to formation of infinite layers (Fig. 5) with molecules stacked along the [0 -1 1] direction. These layers are in turn connected with neighboring layers through the intermolecular N–H···N and C–H···O H-bonds (Fig. 6) to yield a supramolecular architecture sustained by H-bond interactions and π–π interactions. Fig. 7 shows the packing of the molecules along the a axis.

### 2. Experimental

The title compound was prepared by adapting a reported procedure (El-Sherif *et al.*, 2012). (1*Z*)-1-Hydrazinylidene-1,2-dihydrophthalazine hydrochloride (0.299 g, 1.5 mmol) was added to an ethanolic solution of salicylaldehyde (0.122 g, 1 mmol) and sodium acetate (0.204 g, 1.5 mmol). The mixture was stirred well with slight heating for 90 minutes upon which the creamy yellow hydralazone precipitates out. The precipitate was collected by filtration, washed with water (10 ml) and then with 10 ml of ethanol water (1:2) mixture by volume (yield = 66%, 0. 174 g, 0.660 mmol). Single crystals suitable for XRD studies were obatined by recrystallization from a (1:1) mixture by volume of methanol and DMF (m.p: 206 °C).

IR (KBr, υ in cm^-1^): 1613, 3316, 1593, 3100–3200, 1023.^1^H NMR(400 MHz, DMSO-d6, δ in p.p.m.): 10.385 (s, 1H), 8.9 (s, 1H), 8.584 (s, 1H), 8.502 (s, 1H), 7.332–6.902 (m, 8H).

### 3. Refinement

All H atoms on C were placed in calculated positions, guided by difference maps, with C–H bond distances of 0.93 Å. H atoms were assigned *U*_iso_(H) values of 1.2Ueq(carrier). The phenolic O–H distance was restrained to 0.84 (2) Å. The phenolic H atom was found to be disorderd by tautomerism over two positions: partially bonded to O1 and partially bonded to N1 (where the largest Q peak is located after inclusion of extinction correction) with refined occupancies of 0.80 (3) and 0.20 (3) respectively. Partial occupancy of H1 at O1 was also indicated by a rather large *U*_iso_ value for H1A of 0.103 before inclusion of disorder. The *U*_iso_ value for H1B was set to 1.2 times of *U*_eq_ of the N1 atom. H3', located from a difference map, was refined with an N—H distance restraint of 0.88 (2) Å and has a refined *U*_iso_ value of 0.058 Å^2^. Omitted owing to bad disagreement was reflection (0 0 1).

### Crystal data

**Table:**

C_15_H_12_N_4_O	*Z* = 2
*M**_r_* = 264.29	*F*(000) = 276
Triclinic, *P*1	*D*_x_ = 1.381 Mg m^−^^3^
Hall symbol: -P 1	Mo *K*α radiation, λ = 0.71073 Å
*a* = 6.8028 (12) Å	Cell parameters from 1437 reflections
*b* = 8.4263 (13) Å	θ = 2.6–27.4°
*c* = 11.868 (2) Å	µ = 0.09 mm^−^^1^
α = 89.774 (9)°	*T* = 296 K
β = 83.113 (9)°	Block, colorless
γ = 70.356 (8)°	0.25 × 0.20 × 0.20 mm
*V* = 635.62 (19) Å^3^	

### Data collection

**Table:**

Bruker Kappa APEXII CCD diffractometer	2204 independent reflections
Radiation source: fine-focus sealed tube	1623 reflections with *I* > 2σ(*I*)
Graphite monochromator	*R*_int_ = 0.020
Detector resolution: 8.33 pixels mm^-1^	θ_max_ = 25.1°, θ_min_ = 3.2°
ω and φ scan	*h* = −8→8
Absorption correction: multi-scan (*SADABS*; Bruker, 2004)	*k* = −10→10
*T*_min_ = 0.978, *T*_max_ = 0.982	*l* = −13→14
3781 measured reflections	

### Refinement

**Table:**

Refinement on *F*^2^	Secondary atom site location: difference Fourier map
Least-squares matrix: full	Hydrogen site location: inferred from neighbouring sites
*R*[*F*^2^ > 2σ(*F*^2^)] = 0.041	H atoms treated by a mixture of independent and constrained refinement
*wR*(*F*^2^) = 0.117	*w* = 1/[σ^2^(*F*_o_^2^) + (0.0527*P*)^2^ + 0.1254*P*] where *P* = (*F*_o_^2^ + 2*F*_c_^2^)/3
*S* = 1.03	(Δ/σ)_max_ < 0.001
2204 reflections	Δρ_max_ = 0.16 e Å^−^^3^
175 parameters	Δρ_min_ = −0.17 e Å^−^^3^
2 restraints	Extinction correction: *SHELXL97* (Sheldrick, 2008), Fc^*^=kFc[1+0.001xFc^2^λ^3^/sin(2θ)]^-1/4^
Primary atom site location: structure-invariant direct methods	Extinction coefficient: 0.014 (4)

### Special details

**Table:**

Geometry. All e.s.d.'s (except the e.s.d. in the dihedral angle between two l.s. planes) are estimated using the full covariance matrix. The cell e.s.d.'s are taken into account individually in the estimation of e.s.d.'s in distances, angles and torsion angles; correlations between e.s.d.'s in cell parameters are only used when they are defined by crystal symmetry. An approximate (isotropic) treatment of cell e.s.d.'s is used for estimating e.s.d.'s involving l.s. planes.
Refinement. Refinement of *F*^2^ against ALL reflections. The weighted *R*-factor *wR* and goodness of fit *S* are based on *F*^2^, conventional *R*-factors *R* are based on *F*, with *F* set to zero for negative *F*^2^. The threshold expression of *F*^2^ > σ(*F*^2^) is used only for calculating *R*-factors(gt) *etc*. and is not relevant to the choice of reflections for refinement. *R*-factors based on *F*^2^ are statistically about twice as large as those based on *F*, and *R*- factors based on ALL data will be even larger.

### Fractional atomic coordinates and isotropic or equivalent isotropic displacement parameters (Å^2^)

**Table:**

	*x*	*y*	*z*	*U*_iso_*/*U*_eq_	Occ. (<1)
O1	0.8932 (2)	0.3936 (2)	0.68867 (13)	0.0707 (5)	
N1	0.55508 (10)	0.56322 (8)	0.82756 (6)	0.0369 (4)	
H1B	0.6902	0.5244	0.8219	0.044*	0.20 (3)
H1A	0.8225	0.4602	0.7435	0.044*	0.80 (3)
N2	0.43095 (10)	0.68017 (8)	0.91179 (6)	0.0365 (4)	
N3	0.74379 (10)	0.62960 (8)	0.99606 (6)	0.0393 (4)	
N4	0.85914 (10)	0.66505 (8)	1.07304 (6)	0.0438 (4)	
C3	0.72100 (10)	0.18492 (8)	0.47512 (6)	0.0661 (6)	
H3	0.7802	0.1108	0.4127	0.079*	
C2	0.8484 (3)	0.2362 (3)	0.53623 (17)	0.0630 (6)	
H2	0.9931	0.1976	0.5147	0.076*	
C1	0.7630 (3)	0.3452 (2)	0.62990 (15)	0.0455 (5)	
C6	0.5458 (3)	0.40421 (19)	0.66211 (14)	0.0361 (4)	
C7	0.4493 (3)	0.51830 (19)	0.75881 (14)	0.0356 (4)	
H7	0.3032	0.5616	0.7722	0.043*	
C8	0.5352 (2)	0.71106 (18)	0.98897 (14)	0.0322 (4)	
C9	0.4266 (2)	0.84219 (18)	1.07626 (13)	0.0323 (4)	
C10	0.2102 (3)	0.9273 (2)	1.08475 (15)	0.0430 (4)	
H10	0.1302	0.8972	1.0359	0.052*	
C11	0.1156 (3)	1.0551 (2)	1.16484 (16)	0.0504 (5)	
H11	−0.0286	1.1118	1.1698	0.060*	
C12	0.2321 (3)	1.1010 (2)	1.23860 (16)	0.0517 (5)	
H12	0.1664	1.1892	1.2919	0.062*	
C5	0.4208 (3)	0.3510 (2)	0.59677 (15)	0.0472 (5)	
H5	0.2755	0.3907	0.6161	0.057*	
C4	0.5070 (4)	0.2413 (3)	0.50468 (17)	0.0602 (6)	
H4	0.4214	0.2057	0.4628	0.072*	
C15	0.7637 (3)	0.7886 (2)	1.14463 (15)	0.0428 (4)	
H15	0.8421	0.8171	1.1951	0.051*	
C14	0.5434 (3)	0.88579 (19)	1.15182 (14)	0.0363 (4)	
C13	0.4441 (3)	1.0164 (2)	1.23294 (16)	0.0462 (5)	
H13	0.5219	1.0461	1.2832	0.055*	
H3'	0.811 (3)	0.5389 (17)	0.9514 (14)	0.058 (6)*	

### Atomic displacement parameters (Å^2^)

**Table:**

	*U*^11^	*U*^22^	*U*^33^	*U*^12^	*U*^13^	*U*^23^
O1	0.0336 (7)	0.1084 (12)	0.0615 (10)	−0.0118 (7)	−0.0082 (7)	−0.0239 (9)
N1	0.0332 (8)	0.0376 (8)	0.0384 (8)	−0.0096 (6)	−0.0060 (6)	−0.0029 (6)
N2	0.0320 (7)	0.0349 (7)	0.0394 (8)	−0.0067 (6)	−0.0056 (6)	−0.0064 (6)
N3	0.0299 (7)	0.0406 (8)	0.0448 (9)	−0.0073 (6)	−0.0079 (6)	−0.0070 (7)
N4	0.0335 (8)	0.0498 (9)	0.0486 (9)	−0.0121 (7)	−0.0126 (7)	−0.0038 (7)
C3	0.0787 (16)	0.0594 (13)	0.0451 (12)	−0.0035 (11)	−0.0081 (11)	−0.0165 (10)
C2	0.0452 (11)	0.0717 (14)	0.0509 (13)	0.0066 (10)	−0.0017 (9)	−0.0110 (10)
C1	0.0381 (10)	0.0508 (11)	0.0408 (10)	−0.0046 (8)	−0.0090 (8)	−0.0008 (8)
C6	0.0391 (9)	0.0337 (9)	0.0342 (9)	−0.0098 (7)	−0.0067 (7)	0.0018 (7)
C7	0.0307 (8)	0.0368 (9)	0.0388 (10)	−0.0101 (7)	−0.0059 (7)	0.0008 (7)
C8	0.0291 (8)	0.0307 (8)	0.0370 (9)	−0.0097 (7)	−0.0056 (7)	0.0025 (7)
C9	0.0338 (9)	0.0295 (8)	0.0343 (9)	−0.0112 (7)	−0.0057 (7)	0.0041 (7)
C10	0.0344 (9)	0.0438 (10)	0.0486 (11)	−0.0085 (8)	−0.0110 (8)	−0.0025 (8)
C11	0.0394 (10)	0.0477 (11)	0.0534 (12)	−0.0015 (8)	−0.0038 (9)	−0.0080 (9)
C12	0.0587 (12)	0.0429 (10)	0.0470 (12)	−0.0101 (9)	−0.0015 (9)	−0.0102 (9)
C5	0.0475 (11)	0.0526 (11)	0.0445 (11)	−0.0197 (9)	−0.0087 (9)	−0.0034 (9)
C4	0.0703 (14)	0.0607 (13)	0.0509 (13)	−0.0217 (11)	−0.0128 (11)	−0.0132 (10)
C15	0.0368 (10)	0.0492 (10)	0.0469 (11)	−0.0172 (8)	−0.0141 (8)	−0.0013 (8)
C14	0.0392 (9)	0.0364 (9)	0.0368 (10)	−0.0159 (7)	−0.0090 (8)	0.0036 (7)
C13	0.0520 (11)	0.0456 (10)	0.0431 (11)	−0.0171 (9)	−0.0121 (9)	−0.0059 (8)

### Geometric parameters (Å, º)

**Table:**

O1—C1	1.353 (2)	C7—H7	0.9300
O1—H1A	0.8460	C8—C9	1.455 (2)
N1—C7	1.2861 (16)	C9—C14	1.393 (2)
N1—N2	1.3891	C9—C10	1.395 (2)
N1—H1B	0.8600	C10—C11	1.368 (2)
N2—C8	1.3008 (16)	C10—H10	0.9300
N3—C8	1.3654 (16)	C11—C12	1.385 (2)
N3—N4	1.3680	C11—H11	0.9300
N3—H3'	0.886 (9)	C12—C13	1.371 (3)
N4—C15	1.2834 (18)	C12—H12	0.9300
C3—C2	1.367 (2)	C5—C4	1.372 (3)
C3—C4	1.372 (2)	C5—H5	0.9300
C3—H3	0.9300	C4—H4	0.9300
C2—C1	1.383 (3)	C15—C14	1.439 (2)
C2—H2	0.9300	C15—H15	0.9300
C1—C6	1.396 (2)	C14—C13	1.395 (2)
C6—C5	1.394 (2)	C13—H13	0.9300
C6—C7	1.441 (2)		
			
C1—O1—H1A	109.9	C14—C9—C10	119.33 (15)
C7—N1—N2	113.77 (8)	C14—C9—C8	118.79 (14)
C7—N1—H1B	123.1	C10—C9—C8	121.87 (14)
N2—N1—H1B	123.1	C11—C10—C9	120.02 (16)
C8—N2—N1	113.87 (7)	C11—C10—H10	120.0
C8—N3—N4	126.36 (7)	C9—C10—H10	120.0
C8—N3—H3'	118.5 (12)	C10—C11—C12	120.77 (17)
N4—N3—H3'	114.9 (12)	C10—C11—H11	119.6
C15—N4—N3	117.14 (8)	C12—C11—H11	119.6
C2—C3—C4	120.90 (12)	C13—C12—C11	119.97 (16)
C2—C3—H3	119.6	C13—C12—H12	120.0
C4—C3—H3	119.5	C11—C12—H12	120.0
C3—C2—C1	120.24 (17)	C4—C5—C6	121.54 (18)
C3—C2—H2	119.9	C4—C5—H5	119.2
C1—C2—H2	119.9	C6—C5—H5	119.2
O1—C1—C2	118.85 (17)	C3—C4—C5	119.22 (16)
O1—C1—C6	121.08 (15)	C3—C4—H4	120.4
C2—C1—C6	120.06 (17)	C5—C4—H4	120.4
C5—C6—C1	118.02 (16)	N4—C15—C14	124.22 (14)
C5—C6—C7	119.84 (16)	N4—C15—H15	117.9
C1—C6—C7	122.14 (15)	C14—C15—H15	117.9
N1—C7—C6	123.25 (14)	C9—C14—C13	119.80 (15)
N1—C7—H7	118.4	C9—C14—C15	117.65 (15)
C6—C7—H7	118.4	C13—C14—C15	122.52 (15)
N2—C8—N3	125.28 (13)	C12—C13—C14	120.10 (16)
N2—C8—C9	119.19 (13)	C12—C13—H13	120.0
N3—C8—C9	115.53 (12)	C14—C13—H13	120.0
			
C7—N1—N2—C8	172.44 (12)	N3—C8—C9—C10	−175.18 (14)
C8—N3—N4—C15	−1.23 (12)	C14—C9—C10—C11	1.6 (3)
C4—C3—C2—C1	0.6 (3)	C8—C9—C10—C11	−177.04 (16)
C3—C2—C1—O1	179.96 (16)	C9—C10—C11—C12	−0.3 (3)
C3—C2—C1—C6	−0.4 (3)	C10—C11—C12—C13	−1.0 (3)
O1—C1—C6—C5	179.15 (16)	C1—C6—C5—C4	1.2 (3)
C2—C1—C6—C5	−0.5 (3)	C7—C6—C5—C4	−179.45 (17)
O1—C1—C6—C7	−0.1 (3)	C2—C3—C4—C5	0.1 (3)
C2—C1—C6—C7	−179.82 (17)	C6—C5—C4—C3	−1.1 (3)
N2—N1—C7—C6	177.11 (12)	N3—N4—C15—C14	2.9 (2)
C5—C6—C7—N1	174.86 (14)	C10—C9—C14—C13	−1.6 (2)
C1—C6—C7—N1	−5.9 (2)	C8—C9—C14—C13	177.10 (15)
N1—N2—C8—N3	−5.34 (17)	C10—C9—C14—C15	176.45 (15)
N1—N2—C8—C9	174.60 (10)	C8—C9—C14—C15	−4.8 (2)
N4—N3—C8—N2	176.70 (9)	N4—C15—C14—C9	0.2 (2)
N4—N3—C8—C9	−3.25 (16)	N4—C15—C14—C13	178.22 (15)
N2—C8—C9—C14	−173.79 (13)	C11—C12—C13—C14	1.0 (3)
N3—C8—C9—C14	6.2 (2)	C9—C14—C13—C12	0.3 (3)
N2—C8—C9—C10	4.9 (2)	C15—C14—C13—C12	−177.65 (17)

### Hydrogen-bond geometry (Å, º)

**Table:**

*D*—H···*A*	*D*—H	H···*A*	*D*···*A*	*D*—H···*A*
N3—H3′···N4^i^	0.89 (1)	2.31 (1)	3.0181 (14)	137 (2)
O1—H1*A*···N1	0.85	1.89	2.6362 (15)	147
C15—H15···O1^i^	0.93	2.59	3.224 (3)	125

Symmetry code: (i) −*x*+2, −*y*+1, −*z*+2.
